# The Complement System as a Therapeutic Target in Retinal Disease

**DOI:** 10.3390/medicina60060945

**Published:** 2024-06-05

**Authors:** Joshua Ong, Arman Zarnegar, Amrish Selvam, Matthew Driban, Jay Chhablani

**Affiliations:** 1 Department of Ophthalmology and Visual Sciences, University of Michigan Kellogg Eye Center, Ann Arbor, MI 48105, USA; 2 Department of Ophthalmology, University of Pittsburgh School of Medicine, Pittsburgh, PA 15213, USA

**Keywords:** complement system, retinal therapy, choroid, age-related macular degeneration

## Abstract

The complement cascade is a vital system in the human body’s defense against pathogens. During the natural aging process, it has been observed that this system is imperative for ensuring the integrity and homeostasis of the retina. While this system is critical for proper host defense and retinal integrity, it has also been found that dysregulation of this system may lead to certain retinal pathologies, including geographic atrophy and diabetic retinopathy. Targeting components of the complement system for retinal diseases has been an area of interest, and in vivo, ex vivo, and clinical trials have been conducted in this area. Following clinical trials, medications targeting the complement system for retinal disease have also become available. In this manuscript, we discuss the pathophysiology of complement dysfunction in the retina and specific pathologies. We then describe the results of cellular, animal, and clinical studies targeting the complement system for retinal diseases. We then provide an overview of complement inhibitors that have been approved by the Food and Drug Administration (FDA) for geographic atrophy. The complement system in retinal diseases continues to serve as an emerging therapeutic target, and further research in this field will provide additional insights into the mechanisms and considerations for treatment of retinal pathologies.

## 1. Introduction 

The eye is a uniquely immune-privileged organ [1]. Due to this ocular immune privilege, the retina within the eye is not in contact with certain immune mediators in systemic circulation and has a unique host defense which includes the complement system and innate immune cells [2]. The complement system, an imperative component of the immune system, has been shown to be critical in retinal homeostasis and integrity in health and aging. However, dysfunction of the complement system has been implicated in various retinal pathologies [3]. These retinal pathologies include diabetic retinopathy, uveitis, and geographic atrophy (GA) in age-related macular degeneration (AMD) [3,4]. As such, the complement system serves as a unique and promising target for various retinal diseases. 

In this manuscript, we discuss the molecular mechanisms of the complement system and its relationship to the retina in health and aging. We then review current understandings of the complement system in retinal pathologies including pre-clinical cellular, genetic, and animal studies, as well as observations of human retinal pigment epithelium (RPE) cells and murine/primate animal models. We discuss different molecular mediators that may lead to complement activation and subsequent retinal damage. These molecular mediators may serve as potential targets in retinal disease. We then provide an overview of the ongoing and recent clinical trials regarding targeting the complement pathway, including the FILLY trial and the OAKS and DERBY trial. We then discuss Pegcetacoplan (SYFOVRE) and Avacincaptad pegol (IZERVAY), two complement inhibitors approved by the Food and Drug Administration (FDA) for GA. Complement inhibition for retinal pathologies continues to emerge as a field of high clinical interest, and further research will provide additional insights on how to optimize this therapeutic approach for patients. 

## 2. The Complement System 

The complement system is a ubiquitous arm of the innate immune system responsible for modulating immune response, maintaining homeostasis, and clearing debris. Composed of plasma proteins acting as signaling molecules, proteases, and opsonins, the following three distinct pathways of complement activation achieve the same endpoint: the Classical, Alternative, and Mannose-binding Lectin pathways [5,6,7].

The classical complement pathway is initiated when multiple C1q molecules bind to antibody-pathogen complexes. A conformational change results in the cleavage of downstream effectors C4 and C2 to C4b and C2b, which combine to form the C3 convertase. There are similarities between the mannose-binding lectin pathway and the classical pathway. Mannose-binding lectin, a protein similar in function to C1q, is responsible for initiating the complement cascade within the lectin pathway by binding to sugar residues on the surface of specific pathogens. A conformational change in mannose-binding lectin structure potentiates its enzymatic activity. Cleavage of C4 and C2 follows, forming C3 convertase. The alternative pathway does not depend on the presence of pathogens to initiate the cascade; instead, it can be initiated independently when C3 is randomly cleaved to form C3b in a process called ‘tickover’. In this pathway, C3b is complexed with factor B to form C3 convertase, a molecule with a subtly different structure from that seen in the lectin pathway [8,9].

**Figure 1:**
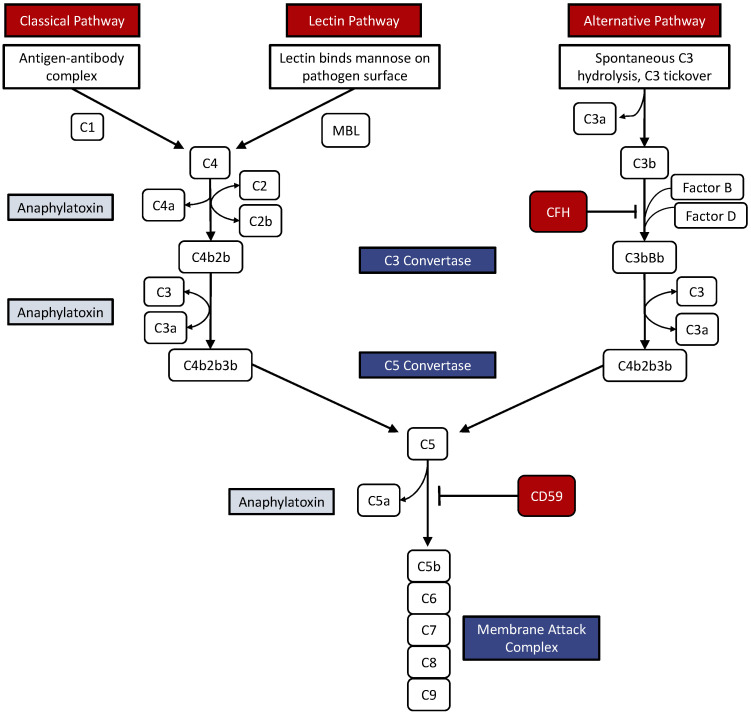
Overview of the complement cascade involving the classical, lectin, and alternative pathways to construct the membrane attack complex. Anaphylatoxins (C3a, C4a, and C5a) may trigger inflammatory responses, while regulatory proteins CFH and C59 may modulate the activity of the complement system.

The formation of C3 convertase arising from any of the complement pathways catalyzes a reaction that yields C3a and C3b molecules. C3 convertase generates C3a, an inducer of an inflammatory response, and C3b, an opsonin. C3b owes its opsonizing capabilities to the presence of a thioester bond that is free to covalently bind with hydroxyl and amine groups on pathogens. C3b-coated pathogens are thus marked for phagocytosis. After C3 convertase is generated, it binds a molecule of C3b and is subsequently called C5 convertase, the structure of which depends on the preceding complement pathway. When arising from the classical and lectin pathways, the structure of C5 convertase is more specifically C4bC2bC3b; otherwise, its structure is C3bBbC3b. This enzyme catalyzes the cleavage of C5 to C5a, an anaphylatoxin, and C5b. The membrane attack complex is formed when C5b binds proteins C6–C9 (Figure 1). This molecule effectively creates a pore in the membrane of the target pathogen to induce lysis [10,11]. Complement proteins do not recognize self-antigens, which help preserve host cells. While the formation of C3b generally results in a feed-forward mechanism that amplifies the immune response, the complement system can be regulated by proteins such as complement factor H (CFH) and complement factor I. Extracellular CFH competes with factor B to inhibit and inactivate C3 convertase, thus dampening the immune response. The intracellular form of CFB cleaves C3 into C3a and C3b [12,13] (Figure 1).

Depicted in Figure 1 are the three pathways of the complement system: the classical, lectin, and alternative pathways. The classical pathway is initiated when an antigen-antibody complex is formed. The C1 complex binds the antigen-antibody complex and triggers the production of C3 convertase. The lectin pathway begins with serum lectin-binding mannose present on the surface of many pathogens. Via mannose-binding lectin, the lectin pathway merges with the classical pathway, as both share the same C3 and C5 convertase enzymes. In the alternative pathway, C3 is spontaneously hydrolyzed on the surface of pathogens and creates a unique C3 convertase and C5 convertase. The C5 convertase is responsible for generating the membrane attack complex, which is capable of lysing cells. Anaphylatoxins (C3a, C4a, and C5a) trigger inflammatory responses, while regulatory proteins CFH and C59 modulate the activity of the complement system. 

## 3. The Retina and the Complement System 

The retina is a tissue comprising specialized cells that function together to convert light signals into electrical impulses and transmit them to the brain [14]. These cells broadly consist of light-sensing photoreceptors, retina ganglion cells with axons that form the optic nerve, and bipolar cells that transmit signals from the photoreceptors to retinal ganglion cells [14,15]. The demise of any of these cells can compromise vision. 

Immune privilege is a protective feature unique to ocular tissue that helps prevent autoimmune reactions and invasion by pathogens [16]. Sir Peter Medawar proposed this term in the 1940s, observing that homologous graft tissue was not rejected when introduced to the anterior chamber of human eyes [17]. Immune privilege suggests that the eye does not communicate with the systemic molecules or immune mechanisms. Factors that grant the eye its immune privilege include physical barriers, the lack of lymphatic-draining ocular tissues, and local immunosuppressive proteins [16]. Despite the eye’s immune privilege, the complement system actively surveils ocular tissues, such as the retina, ready to intervene in the rare event of infection or stress [16].

Systemically, complement is largely produced by the liver in an inactive form as an acute-phase reactant. The retina, however, may produce complement locally [18]. The retinal pigment epithelium (RPE) and retinal ganglion cells have been shown to produce C1q, C2, and C4 constitutively. CFH, complement factor B, C5, and C3 have also been identified in the human retina [19,20,21]. During synaptogenesis early in life, astrocytes act on retinal ganglion cells to produce C1q, which can opsonize certain synapses and mark them for destruction [22]. Astrocytes are also pivotal in organizing retinal vascular development. Their activity is modulated by microglia through the C3 axis. Deficiency of C3 in mouse models leads to avascular regions in the retina. C1q−/−, Mbl−/−, Fb−/−, C3−/−, and C5−/− knockout mice exhibit inner-retina thinning compared to control, while C3−/− knockout models also demonstrate swelling of photoreceptor outer segments and Bruch membrane thickening [23,24]. C1q levels dramatically increase with age, suggesting a possible role for complement dysregulation in neurodegenerative diseases [25]. Local factors work synchronously during development and to achieve retinal homeostasis during natural aging.

## 4. Activation of the Complement System in Retinal Disease

### 4.1. Age-Related Macular Degeneration

**Figure 2:**
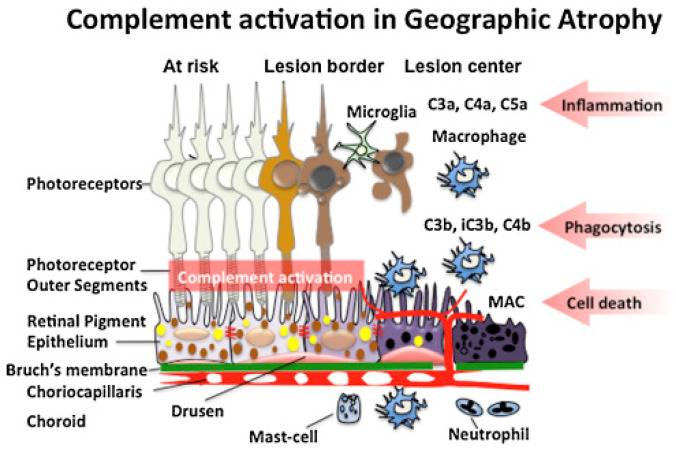
Complement activation in geographic atrophy. The deterioration of retinal pigment epithelium (RPE) in geographic atrophy (GA) allows for additional complement to access the retina. Complement activation leads to the formation of the membrane attack complex, thus leading to destruction and cell death of photoreceptors and RPE in GA. Figure reprinted with permission from Campagne et al. [41] under Creative Commons Attribution 4.0 International License (https://creativecommons.org/licenses/by/4.0/legalcode.en, accessed on 1 March 2024).

AMD is a progressive, degenerative retinal disease typically seen in individuals of advanced age [26,27]. It is characterized by abnormalities of macular photoreceptors, RPE, Bruch membrane, and choroid, resulting in loss of central vision. Depending on the stage of the disease, eyes with AMD present with macular drusen, RPE atrophy, and/or choroidal neovascularization (CNV). AMD classification is dependent primarily on the presence or absence of CNV [28]. If CNV is present, the disease is considered clinically exudative AMD, while lack of CNV is considered non-exudative [29]. Formal classification systems consider drusen >63 μm but <125 μm without pigmentary changes to be early stage AMD. Larger drusen or pigmentary changes are considered intermediate AMD. Late AMD is an exudative disease or GA (large-scale demise of macular RPE) [30]. GA develops following long-standing inflammation and is mediated by the complement system. A depiction of the molecular mechanisms involved in GA is shown in Figure 2. Vision loss occurring in AMD patients occurs due to the dysfunction of photoreceptors. It is believed that intrinsic and extrinsic oxidative stressors are at the core of this dysfunction [31]. Due to the high metabolic demand, exposure to light radiation, and the presence of polyunsaturated fatty acids, the retina is particularly vulnerable to oxidative stress [32]. This helps explain why smoking, which worsens oxidative stress by generating reactive oxygen species, is a risk factor for AMD [33,34,35]. These oxidative factors lead to the disruption of the RPE and drusen accumulation. Excessive drusen and cellular waste accumulation can initiate abnormal immune responses, leading to inflammation [36]. As explained in a subsequent section, the complement system plays a significant role in retinal diseases and AMD [37,38]. Chronic inflammation can worsen RPE cell function and eventually cause outer retinal, RPE, and choriocapillaris cell death [39]. This form of atrophy leads to the sharply demarcated lesions of GA. Management of early and intermediate AMD includes counseling, smoking cessation, consuming leafy vegetables, use of AREDS vitamins, and clinical monitoring for signs of CNV, such as metamorphopsia and central scotoma. Exudative AMD is typically treated with anti-vascular endothelial growth factor injections [40], while GA treatments will be discussed below.

**Figure 3:**
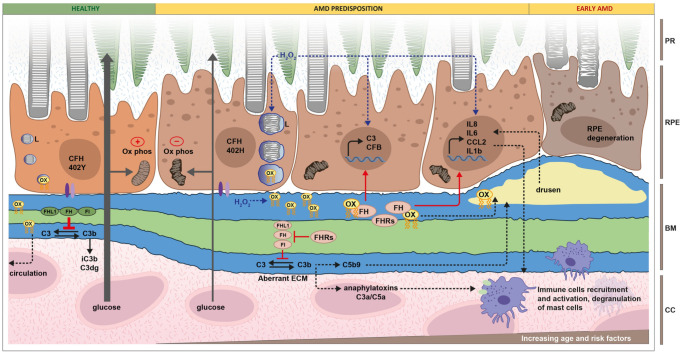
Pathologic changes that precede age-related macular degeneration (AMD). The green section indicates that healthy eyes without CFH Y402H mutations can freely regulate transport of oxygen and other nutrients from the choroidal circulation (circulation) through the blood-retinal barrier and to the RPE and photoreceptors. In eyes with this CFH Y402H mutation, there is a predisposition for AMD (yellow section). The RPE cells exhibit decreased oxidative phosphorylation (ox phos) capacity. In conjunction with natural aging, there is a disruption in transport through the Bruch membrane. Unchecked complement activation occurs depending on the severity of the mutations and the degree of extracellular matrix (ECM) disruption. Oxidative stress induces RPE cell damage, further promoting a pro-inflammatory state by causing cytokine release (IL-6, IL-8). In early stage AMD (red), there is an accumulation of oxidized lipoproteins (drusen) that form in the Bruch membrane. There are degenerative changes in macular RPE cells and photoreceptors. Figure reprinted with permission from Armento et al. [48] under Creative Commons Attribution 4.0 International License (https://creativecommons.org/licenses/by/4.0/legalcode.en, accessed on 1 March 2024).

**Figure 4:**
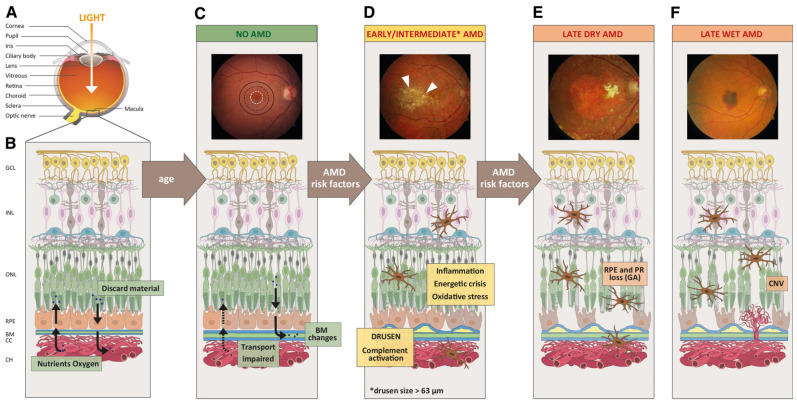
Natural aging and the progression to late-stage AMD. This figure demonstrates the key stages of AMD progression, beginning with an overview of ocular anatomy (**A**,**B**), natural aging processes (**C**), early/intermediate AMD (**D**), late-stage non-exudative (dry) AMD (**E**), and finally late-stage exudative (wet) AMD (**F**). In natural aging, changes in the Bruch membrane (**C**) impair the exchange of nutrients for waste, damaging local structures such as the RPE. Inflammatory processes take hold, and the complement system is activated (**D**). In late-stage disease, there is either widespread RPE atrophy (geographic atrophy) (**E**) or else CNV network formation (**F**). Figure reprinted with permission from Armento et al. [48] under Creative Commons Attribution 4.0 International License (https://creativecommons.org/licenses/by/4.0/legalcode.en, accessed on 1 March 2024).

GA is characterized by large-scale RPE destruction in the macula. These lesions partly develop due to the activation of anaphylatoxins and the membrane attack complex, which targets local cells, including the photoreceptors and the RPE. Other immune cells are recruited to the site to phagocytose cell fragments. The pathophysiological underpinnings of AMD that lead to these findings are not completely understood. Age-related disruption in the Bruch membrane has been proposed as a possible factor (Figure 3) [42,43,44]. In these studies, lipid accumulation in the Bruch membrane increased with age, impairing the transport of lipophilic waste from the photoreceptors/RPE into the choroidal circulation [42,45]. The lipid aggregates undergo chemical modifications, including oxidation, that induce a pro-inflammatory state. These molecules are components of drusen seen in AMD (Figure 4) [46,47,48]. 

The complement system is thought to play a considerable role in AMD pathogenesis [49]. Firstly, individuals who are homozygous for the rs1061170 polymorphism in CFH (Y402H substitution) are associated with a 5.52× increased likelihood of developing AMD [50,51,52]. The CFH gene is found on chromosome 1q31, and its protein product is a negative regulator of the complement cascade, as described above [50]. Genome-wide association studies have identified that polymorphisms in genes encoding complement factor B, complement factor H-related protein 3, complement factor I, C2, and C3 are associated with AMD in varying capacities [53,54,55]. 

Oxidative stress caused by natural aging and environmental factors, such as smoking and poor diet, likely activates the complement system. This pro-inflammatory environment may be responsible for patterns observed in complement expression with aging. Complement regulatory proteins such as CFH are produced in greater concentrations by RPE cells under normal physiologic conditions than complement effector proteins such as C3. This dynamic is reversed in the aging retina, such that elevated levels of C1q and C3 proteins are observed in mouse models [56,57]. Notably, CFH, C3, and terminal complement proteins C5-C9 have been identified in drusen deposits in AMD, while C5 was isolated from RPE cells adjacent to drusen [38]. Increased levels of complement proteins C3d and complement factors B and D were observed in the plasma of patients with AMD, suggesting a possible systemic overactivation of the complement cascade in this disease [58].

Mouse models of CNV have provided insights into the relationship between the complement system and exudative AMD. Damaging the Bruch membrane using laser photocoagulation induces CNV in mice [59]. Studies by Bora et al. indicated that CNV requires complement. In one study, C3−/− laser-treated mice failed to induce CNV, and elevated levels of terminal complement were found in the CNV networks in complement-sufficient mice [60]. Subsequently, their group found that C5−/− mice had blunted CNV formation compared to complement-sufficient counterparts. They also suggest that the alternative pathway is likely the key aspect of the complement cascade involved in CNV development [61]. Nozaki et al. found C3aR- and C5aR-deficient mice to also exhibit decreased CNV formation [62].

Given the prominent role of complement dysregulation in AMD, therapeutics aimed at inhibiting the complement cascade have demonstrated value in preventing the progression of late-stage AMD. Intravitreal pegcetacoplan (Syfovre; Apellis Pharmaceuticals, Inc., Waltham, MA, USA) was recently approved in 2023 in the United States for treating GA. As a C3 inhibitor peptide, pegcetacoplan acts early in the complement cascade to prevent the activation of C3 convertase and the eventual production of the membrane attack complex [63]. Avacincaptad pegol intravitreal solution (Izervay; Astellas Pharma Inc., Tokyo, Japan) was also approved in 2023 in the United States as a treatment for GA. Its mechanism of action differs slightly from that of pegcetacoplan, in that it is a pegylated RNA aptamer that binds C5 to prevent the activation of C5 convertase [64]. A more in-depth discussion of AMD treatments that target the complement cascade will be carried out in a separate section.

### 4.2. Complement in Uveitis

Uveitis is a potentially blinding inflammatory disease affecting the choroid, ciliary body, and iris that develops idiopathically or as a response to viral infections or systemic inflammatory/autoimmune conditions. The role of complement activation in uveitis has been well studied using animal models in a process called experimental autoimmune uveitis (EAU). The initial models of EAU were developed by injecting rats with rhodopsin, producing uveoretinitis of dose-dependent severity [65]. Since then, EAU has been achieved via immunization of rodents with a number of other proteins, such as recoverin and phosducin [66]. The inflammatory processes in EAU are T-cell mediated, but T-cell activation may, in turn, be modulated by complement [67]. For instance, the cell surface receptor CD55 regulates T-cells by inhibiting C3 and C5 convertase and dampening the production of anaphylatoxins C3a and C5a. Mice lacking the gene encoding CD55 demonstrated altered T-cell responses in EAU [68,69]. Read et al. found that mice deficient in C3 were less likely to develop EAU overall and had less severe EAU than controls [70]. Inhibiting C5 cleavage and terminal complement/membrane attack complex activation significantly reduced disease progression and severity in mice EAU models [21]. In a similar vein, C9 inhibition via AAV-mediated delivery of soluble CD59 (a C9 inhibitor) reduced the membrane attack complex and NLRP3 inflammasome activation, and it also decreased disease severity [71]. Targeting the complement system with therapeutic strategies may prove beneficial in treating uveitis.

### 4.3. Complement in Diabetic Retinopathy

Diabetic retinopathy (DR) is a common cause of vision loss that affects around one in twenty Americans as of 2021 [72]. The pathogenesis of DR is multifactorial and involves inflammatory and immune processes. Elevated blood glucose damages retinal vessel endothelial cells via advanced glycation end product formation and increases endothelial permeability, causing microaneurysm, hemorrhage, exudation, and ischemia. Chronic inflammation can result in diabetic macular edema. Ultimately, there can be angiogenesis of aberrant blood vessels and subsequent hemorrhaging, referred to as proliferative DR (PDR). 

Several studies point to dysregulation of the complement system in DR. Gerl et al. isolated C3d and C5b–C9 complexes from the choriocapillaris of patients with DR but notably did not find C1q, C4, or mannose-binding lectin at the time [73]. In conjunction with the data indicating that hyperglycemia interferes with the normal function of free and cell membrane-bound complement, it has been theorized that the alternative pathway is spontaneously activated in these patients [74]. García-Ramirez et al. later found elevated levels of C4b in the vitreous of PDR eyes, suggesting the alternative and classical pathways may contribute [75]. Huang et al. propose that immunoglobulin-associated exosomes may induce the classical complement cascade in diabetic eyes, leading to retinal vascular damage [76]. In the section on exudative AMD pathogenesis, we discussed that complement may be pro-angiogenic in the setting of CNV. Briefly, in C3 knockout mice, there was a poor angiogenic response to laser-induced injury [59]. Studies report increased C3, C4b, C9, factor B, and CFH in the vitreous of eyes with PDR [4,75]. Decreased levels of CD55 and CD59, regulators of the complement cascade, have been reported in diabetic eyes [77]. The expression of CFH is upregulated in microglia in diabetic eyes, perhaps indicating a form of negative feedback in late-stage DR [4]. Furthermore, several polymorphisms of CFH, C5, and CFB have been associated with DR [78,79,80]. 

## 5. Targeting Complement Pathways in Retinal Disease

### 5.1. In vitro and Genetic Studies

In vitro studies targeting the complement pathway in the retina have established a proof-of-concept framework for developing useful pharmaceuticals for retinal disease. In this section, we report the various in vitro and genetic studies on complement inhibition in retinal diseases. 

C1q, a subcomponent of the C1 complex and initiating molecule of the classical pathway, has garnered significant research attention [81,82]. An in vitro RPE cell culture model mimicking drusen formation in C1q-depleted human serum demonstrated significantly less C5b-9 activation compared to control serum, suggesting utility of C1q inhibition in preventing downstream complement activation [83]. Interestingly, the effects of C1q absence or inhibition are ambiguous: In an in vitro rat explant model of degenerative retinal disease, designed to study the effects of C1q on retinal health after acute injury, cells receiving supplemental C1q demonstrated significantly increased ganglion cell survival compared to cells treated with a C1-inhibitor or receiving no supplementation [84]. Their experiment used Berinert, a human C1-esterase inhibitor (C1-inh) [85]. Notably, the cells received serum-lacking factors necessary for further complement activation, such as C1r/C1s, so the effect of supplemental versus inhibited C1q on cell survival appear to be a direct one rather than a classical component-mediated one. Work on C1q inhibitors as a target for retinal disease, therefore, must contend with the possibility of negating direct or indirect benefits of the complement pathway. 

TNT009, an anti-C1s antibody, has demonstrated promising in vitro results, significantly decreasing HLA antibody-triggered complement activation [86,87]. It has entered clinical trials for other complement-mediated disorders, like cold agglutinin disease, under the name BIVV009, but it has not seen specific use for retinal pathology (NCT02502903). Its preliminary results in other complement-mediated disorders suggest possible future utility in the retina.

There is a paucity of data or investigations on mannose-binding lectin (Mbl) as a specific complement target in retinal disease, as investigations have thus far preferentially targeted downstream pathways common to the classical and lectin pathways when not directed at C1. Potential shared targets prior to convergence with the alternative pathway include C4, C4a, C2, C2b, C4b2b (C3 convertase), C3, C3a, and C4b2b3b (C5 convertase). Of these targets, C3 and C3a have garnered the most research attention and also have relevance in the alternative pathway prior to the convergence of all three pathways.

C3 and related protein experimentation in vitro has yielded several key insights. First, and not specific to ocular-focused in vitro studies, C3 inhibition prevents inflammatory cytokine-mediated complement activation and reduces inflammation [88,89]. In RPE cells from patients with AMD and the Y402H polymorphism in factor H, inhibition of C3 processing with a compstatin ameliorated disease by restoring lysosomal function [90]. In an in vitro study of embryonic chick retinal cells, C3a appears to have a role in inducing retina regeneration via STAT3 activation [91]. Additionally, in a human fetal RPE cell model, C3a induced formation of sup-RBE deposits and impaired extracellular matrix turnover, with both effects being mediated by the ubiquitin proteasome pathway [92]. Notably, formation of these deposits was prevented by adding a C3a receptor (C3ar) antagonist. Gorham et al. describe a novel human RPE cell-based model for future in vitro studies, validated using compstatin inhibition of C3 activity [93]. Their work is useful and important in developing future therapeutic targets, as it may allow for higher throughput experimentation not requisite on animals, which may be time-intensive to develop, and because compstatins, which are often-studied proteins of clinical interest for the complement pathway, have specificity for human and primate C3 only. These studies will be of great interest given C3′s historic focus on developing complement-directed therapeutics as a central part of the cascade.

There have been several key in vitro results discovered after investigating C5, another central cog in the complement cascade. In non-retinal in vitro studies, C5 inhibition demonstrates neuroprotectivity [94]. One in vitro study on human RPE cells showed that C5 depletion and C5a receptor (C5ar) inhibition suppressed priming signals for inflammasome activation [95]. In another in vitro human RPE cell study, this time on rhegmatogenous retinal detachment associated with choroidal detachment, introduction of C5a led to increased viability and aggravated inflammation in cells while also leading to downstream activation of a number of pro-inflammatory factors, such as tumor necrosis factor alpha (TNF-α) and IL-6 [96]. A combined in vitro-in vivo study on uveoretinitis demonstrated suppressed disease with blockage of C5 cleavage, likely related to activity of the inhibitory myeloid CD200 receptor [21]. There are relatively few in vitro studies directly studying retina effects of C5-inhibition or absence, but there is significant ongoing research involving animal and clinical models.

There are fewer in vitro studies on factors B, D, H, and I than other components of complement. Sparse in vitro data on factor B suggest that its induction by TNF-α is able to be blocked by treatment with 5-aminoimidazole-4-carboxamide riboside, an AMP-dependent kinase activator, in an RPE cell model [97]. Other studies corroborate the role of TNF-α in upregulating factor B production and add that it downregulates factor H production [19,98]. In vitro studies on erythrocytes show that inhibition of factor B is effective in inhibiting alternative pathway activity [99,100].

Factor D inhibition is another selective approach for blockade of the alternative pathway. In vitro data note that its inhibition is effective in blocking the alternative pathway while preserving other paths of the cascade, preserving its beneficial roles in bacterial lysis and cleavage, resulting in a reduced risk of infections compared to a more central inhibition [101,102,103]. In vitro factor D studies focusing on the retina are sparse, but development of Danicopan, a factor D inhibitor included in in vitro studies, has demonstrated its binding properties to melanin, which is important for drug dosing to melanin-containing tissues like the choroid and retina [104].

Factor H dysfunction has been implicated in AMD pathology in in vitro studies. One study of in vitro competition assays demonstrated that, when FHR-4, a protein linked to AMD pathogenesis, is present in high concentrations, factor H is outcompeted for C3b binding, preventing factor H-mediated complement inactivation [105]. The introduction of factors H and I, other key complement regulatory proteins, represents other possible targets of complement-mediated disorders but has largely been studied in vivo to date.

### 5.2. Animal Studies

Murine studies have built off in vitro results on various complement proteins and pathways. In a large in vivo study covering key components in each complement pathway, Mukai et al. compared amplitudes of electroretinograms (ERGs) and retinal layer thickness on spectral domain OCT (SD-OCT) between young (6-week-old) and adult (6-month-old) mice to analyze the complement system’s role in maintaining retinal integrity [23]. They compared wild-type mice to separate C1q, Mbl, factor B, C3, and C5 knockout strains. Functional abnormalities correlating with thinning of the inner retina were detected in all adult knockout mice compared to young knockout mice. Wild-type adult mice had relatively preserved retinal functions compared to young mice, indicating that complement may play a role in maintaining structural and functional retinal integrity. These in vivo results suggest that caution need be taken when considering pharmaceuticals targeting these pathways. Subsequent results on inhibition and knockout have, at times, urged caution in inhibiting a key physiologic pathway of immunity; however, they have also produced very promising results that have paved the way for clinical trials and approved pharmaceuticals. 

In the classical pathway, C1 and C1 subcomplexes have been a popular area of study. In a streptozotocin-induced diabetes rat model, C1-inh decreased retinal vascular permeability [106]. Inhibition of plasma kallikrein (PK), which is physiologically inhibited by C1 inhibition, by a selective PK inhibitor, ASP-440, also decreased vascular permeability. Conversely, intravitreal injection of PK resulted in increased retinal thickness on optical coherence tomography (OCT) in diabetic rats, an effect also observed in nondiabetic rats (but to a lesser extent). Notably, PK is also able to cleave C3 and activate complement, merging with the alternative pathway [107]. 

In a mouse model, C1q knockout strains demonstrated decreased photoreceptor cell death, diminished microglia recruitment, and improved visual function [108]. In the same model, intravitreal but not systemic delivery of a C1q inhibitor slowed retinal degeneration following photo-oxidative damage, suggesting an importance in the delivery method when targeting the complement system. Following strong in vitro and murine studies on C1q, ANX007, a C1q-directed antibody, was tested in a non-human primate model and was well tolerated and successfully directed to key sites of neurodegenerative disease in the retina, supporting the merit of future studies in humans [109].

Due to its central role in multiple steps of the complement cascade, there has been extensive pre-clinical animal work targeting C3. An early investigation in a cynomolgus monkey model of AMD used a compstatin C3 inhibitor, demonstrating suppression and reversal of drusen formation [110]. Given promising preliminary work, subsequent work using compstatin-based inhibitors of C3 on primates have analyzed pharmacokinetics and tissue penetrance, demonstrating favorable results [111].

Despite these promising results, other studies, such as that by Hoh Kam et al., have urged caution in complete inhibition of C3 for treatment of AMD [24]. While their work corroborated that uncontrolled activation of C3, investigated through mice deficient in complement factor H (CFH), was associated with negative effects on retinal aging, including amyloid β deposition and inflammation, complete absence of C3 also had deleterious effects on retinal aging. Later studies involving complete C3 ablation have described a positive impacts of deletion, including decreased retinal degeneration [112,113]. Furthermore, exogenous delivery of C3 to murine RPE produces functional and anatomic changes resembling AMD [114]. In spite of a lack of total agreement on C3 inhibition on retinal disease, the promising data in animal models appear to outweigh the negative data. Additionally, it should be noted that pharmacologic inhibition in response to disease at a specific point in life likely carries different implications for retinal health than a complete lack of complement activity throughout life and key developmental stages [115]. Additionally, as with other complement targets, delivery target and route is important: delivery of an adeno-associated virus binding to C3-based opsonins basal to the RPE (subretinal) resulted in complement inhibition in mice, while a cassette directed to retinal ganglion cells (intravitreal) did not produce the same inhibition, likely due to inefficient transportation across the RPE [116].

C3 subcomplex and related protein work has included investigations on C3a, C3ar, and C3 convertase. In a mouse model, C3a supplementation upregulated immunoproteasome activity, implicating possible proteasome activity in the development of AMD [117]. Later, investigative work on a pharmacologic inhibitor of C3ar in mice, intending to negate the deleterious effects of C3a, showed alleviated neuroinflammation and improved visual function with inhibition [118]. Additional work on C3ar, however, has remained ambiguous. In two mice knockout studies, one demonstrated that C3ar-knockout mice had early onset and progressive retinal degeneration, while another showed that C5ar knockouts, but not C3ar knockouts, had attenuated retinal damage after light exposure compared to wild-type mice [119,120]. These mixed results may be partially responsible for a paucity of human trials focusing on C3a or C3ar.

There have been a number of animal studies on C5 and related proteins as a possible central complement target. In a mouse model, an anti-mouse C5 antibody inhibited of choroidal neovascularization (CNV) induced by laser photocoagulation, was found to have reduced production of C5a and had no definite signs of toxicity [121]. Notably, it targeted a different epitope than eculizumab, a C5 inhibitor with inconclusive results in human trials on GA. In rodent models, although in a model of glaucoma, it was observed that C5 inhibition has prevented deterioration of retinal function, as well as loss of retinal ganglion cells, cones, and photoreceptors, and it has also helped to preserve optic nerve function [122,123]. In a murine model of autoimmune uveoretinitis, monoclonal antibody treatment against C5 cleavage, delivered systemically and locally, resulted in significantly reduced disease scores compared to controls [21]. Notably, however, other animal work has emphasized that C3 may be a more advantageous target than C5, as inhibition of it blocks formation of sub-RPE deposits, while genetic C5 ablation is not able to similarly prevent formation of these deposits, potentially due to increased activation of C3 in response to complement dysregulation [124]. 

Generally, inhibition of C5a or C5ar have not produced strong results. Systemic administration of an anti-C5a antibody was insufficient to block the development of AMD-like pathologies in mice [125]. The authors suggest that targeting other components of the complement cascade, such as C3a or MAC, may be more important in treating AMD, potentially because C5a inhibition is not able to block sub-RPE deposit formation. C5ar targeting has been slightly more promising than targeting C5a itself, as murine data have demonstrated C5ar’s key role in recruiting microglia after light damage, its involvement in subretinal fibrosis, and its central role in maintenance of normal retinal function and structure [119,120,126]. A study of a murine model of retinopathy of prematurity, however, demonstrated a potential downside of C5 inhibition, as C5ar-deficient mice demonstrated increased neovascularization [127]. 

Other animal studies on the complement cascade have included investigations on CD59 and factors B, D, H, and I. An in vivo rat study examined the effects of alternative pathway inhibition by human plasma-purified complement factor H (CFH) in a model of laser-induced CNV [128]. In their model, intravitreal CFH injection suppressed formation of new CNV while improving the status of existing CNV. Another investigation of CNV in a murine model examined the effects of CD59, an inhibitor of MAC formation, on CNV [129]. The recombinant membrane-targeted CD59 similarly inhibited growth of CNV while reducing the size of already-developed CNV. 

Factor B deletion in a mouse model of AMD and Doyne honeycomb retinal dystrophy/malattia leventinese resulted in normalization of dysregulated complement pathways, while pharmacologic inhibition resulted in a reduction in sub-RPE deposits [130]. Interestingly, the researchers found that the effects on sub-RPE deposits by pharmacologic inhibition differed by sex, with male mice having fewer sub-RPE deposits than age-matched females but no effect of factor B inhibition on sub-RPE deposits. The reasons underlying the different responses between sexes remains unclear, but there are data describing differences in blood complement levels between healthy females and males, with one study describing higher levels of factor B in females than males [131]. There is, however, evidence of a positive role of factor B in neovessel clearance, again urging caution when developing therapeutics targeting this arm of the complement cascade [132].

Animal research focusing on inhibition of factor D has produced promising results, leading to development of several pharmaceuticals that have advanced to clinical trials. In mice, factor D knockout strains demonstrated significant photoreceptor protection after constant light exposure compared to controls [133]. Further studies have built on this promising finding by characterizing pharmacokinetic and pharmacodynamic profiles in cynomolgus monkeys, and work has supported advancement to clinical trials in humans [134,135,136]. The development of danicopan, a factor D inhibitor in ongoing clinical trials, is a promising next step from these pre-clinical studies [137].

Factor I has had several pre-clinical studies, leading to development of GT005, an adeno-associated virus gene therapy promoting factor I expression [138,139]. Its results include a significant and dose-dependent reduction in CNV with no adverse systemic effects. Table 1 summarizes the results of the pre-clinical results in complement inhibition in the retina.

### 5.3. Clinical Trials

Ongoing and recent clinical trials targeting the complement pathway have developed from the framework and results of pre-clinical studies, and they have similarly targeted facets of each pathway (Table 2). In the classical pathway, Cinryze, a C1 esterase inhibitor used for hereditary angioedema, underwent a phase 1b safety and proof-of-concept clinical trial for patients with NMO (NCT01759602) [142]. Cinryze demonstrated promising preliminary results in safety and efficacy in the 10 patients tested but has not had any further clinical trials since the study’s completion in 2014.

As with pre-clinical studies, C3 has been a popular target in clinical trials. APL-2 (SYFOVRE, Apellis Pharmaceuticals), a pegylated peptide/second generation compstatin inhibiting C3 cleavage, received FDA approval in 2023 for GA, a landmark event as the first FDA-approved treatment for GA [63,115]. Other C3-directed therapies in ongoing or recent clinical trials include NGM621 (NGM Bio, San Francisco, CA, USA, NCT04014777/NCT04465955) and POT-4 (Potentia Pharmaceuticals, Louisville, KY, USA, NCT01603043) [115,143]. NGM621 is a monoclonal antibody that binds to C3 and prevents cleavage to C3a and C3b. It demonstrated safety and tolerability in a phase 1 trial for GA secondary to AMD which concluded in 2020, and it recently completed a phase 2 trial on 320 participants. It represents another promising C3-targeted therapeutic, and APL-2′s approval may facilitate approval for it and other related pharmaceuticals. POT-4 is a compstatin analog binding C3b and C3c with less favorable results than APL-2 or NGM621 [144,145]. The first complement inhibitor that underwent trials for AMD, it demonstrated favorable results in a phase 1 trial before being terminated in phase 2.

There have been a number of recent and ongoing clinical trials on C5-directed therapeutics. Avacincaptad pegol (Izervay, previously Zimura, Astellas Pharma), a C5 inhibitor, was the second pharmaceutical to be approved by the FDA for GA, another landmark in complement therapeutics and GA treatment [64,146]. Beyond its approval for GA, it has been investigated in trials for use in patients with polypoidal choroidal vasculopathy (NCT03374670, phase 2 completed), autosomal recessive Stargardt disease (NCT03364153, phase 2 recruiting), nAMD along with anti-VEGF therapy (NCT03362190, phase 2 completed), and an open-label extension for GA (NCT05536297, phase 3 recruiting). The diversity of pathologies under current investigation for treatment with avacincaptad pegol/Zimura highlights the diversity of conditions theoretically treatable with complement-targeted therapeutics. Other C5 inhibitors include Eculizumab (NCT00935883) and LFG316/Tesidolumab. Eculizumab is approved for various other complement-mediated conditions, including paroxysmal nocturnal hemoglobinuria and atypical hemolytic uremic syndrome [145]. It was well tolerated in 30 patients in a phase 2 trial in patients with GA, but it did not significantly decrease the growth rate of GA, the primary endpoint of the study [147]. Notably, its delivery was intravenous, while other more successful approaches, like Avacincaptad pegol, have been intravitreous, suggesting that where the drug is delivered may be just as important as the formulation itself. However, clinical trials on LFG316 (Tesidolumab, Novartis. Basel, Switzerland), another C5 inhibitor approved for other complement-mediated disorders, for treatment of GA secondary to AMD (NCT01255462) and uveitis (NCT01526889) did not result in significant amelioration of pathology regardless of intravenous or intravitreal delivery, and further trials were not pursued after phase 2 trials were completed [148]. 

Clinical trials on complement-directed therapeutics have been much less frequent on non-C3 or C5 targets. Targets have included factor B, factor D, factor I, and properidin. IONIS-FB-LRx (Ionis Pharmaceuticals, Carlsbad, CA, USA) is an antisense inhibitor of complement factor B currently in a phase 2 trial for treatment of GA (NCT03815825). In a phase 1 trial including 54 healthy volunteers, it was well tolerated and resulted in a reduction in factor B levels [149]. 

FCFD4514S (Lampalizumab, Genentech, San Francisco, CA, USA) is a factor D inhibitor that underwent several trials for GA (NCT02288559). While tolerated well, it had no apparent therapeutic benefit, with no significant reduction in GA enlargement compared to sham therapy over 48 weeks [150]. Genentech terminated phase 3 trials. ALXN2040 (Danicopan, AstraZeneca, Macquarie Park, NSW, USA) is another factor D inhibitor currently in a phase 2 trial for treatment of GA (NCT05019521). Results have not yet been posted in regard to GA, but it has shown promising results in phase 3 trials for patients with paroxysmal nocturnal hemoglobinuria when combined with a C5 inhibitor [151]. 

CLG516 (Novartis), a properidin inhibitor, progressed to phase 2 trials for treatment of GA (NCT02515942). It was administered as a monotherapy and in combination with LFG316; however, it failed to halt GA progression, and no further investigations were undertaken after the phase 2 trial [152].

GT005 (Gyroscope Therapeutics/Novartis) is a novel adeno-associated virus gene therapy promoting complement factor I production, downregulating complement activity [62]. After promising pre-clinical studies, several clinical trials for treatment of GA were initiated, but development was discontinued in 2023 following data from the phase 2 HORIZON trial (NCT04437368, NCT05481827, NCT04566445, NCT03846193). Table 2 summarizes the clinical trials targeting the complement system for retinal disease.

## 6. Clinically Available Inhibition Therapy in Geographic Atrophy

Geographic atrophy (GA) is an irreversible, advanced form of dry AMD [31,153]. Unlike neovascular AMD, which is characterized by irregular neovascularization and fluid leakage, GA is characterized by atrophy of outer retinal tissue, RPE, and choriocapillaris, leading to progressive vision loss [154,155,156]. Lesions typically begin in the perifoveal region and expand to the fovea over time, which leads to central scotomas and decreased visual acuity. The rate of disease progression varies greatly between individuals, but, on average, GA progresses at a rate of 1.78 mm2 per year, with a median predicted progression to legal blindness of 6.2 years [154,157]. 

GA affects approximately 5 million people worldwide and is estimated to affect 10 million people by 2040 [158]. Age is one of the strongest risk factors for GA, and the mean age for a patient with GA is 79 years [159]. Prevalence of disease increases [31] to 2.91% in patients greater than 80 years and 11.29% in patients greater than 90 years [160]. Smoking, both current use and former use, increases the risk for developing GA [161]. Additional risk factors include family history and genetic mutations in the complement system [155,162]. 

### 6.1. Imaging

**Figure 5:**
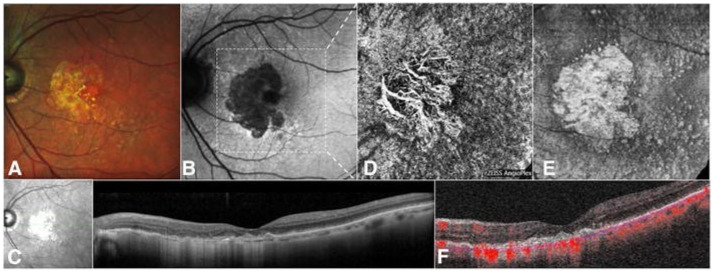
Geographic atrophy visualized using different imaging modalities. Box (**A**) demonstrates multicolor imaging showing reflectance in different wavelengths in geographic atrophy. Box (**B**) demonstrates fundus autofluorescence, showing geographic atrophy as sharply demarcated dark regions. Box (**C**) shows infrared reflectance with an adjacent optical coherence tomography (OCT) B-scan of the central fovea, showing the loss of outer retinal layers and retinal pigment epithelium. Box (**D**) demonstrates en face OCT angiography, showing loss of flow at the choriocapillaris within the lesion. Box (**E**) demonstrates en face OCT, showing hyper-reflectance at the choriocapillaris layer due to backscattering. Box (**F**) demonstrates an OCT angiography B-scan of the central fovea showing loss of flow in the choriocapillaris. Reprinted with permission from Sacconi et al. [163] under Creative Commons Attribution-NonCommercial 4.0 International License (https://creativecommons.org/licenses/by-nc/4.0/legalcode.en, accessed on 1 March 2024).

Advancements in imaging and multimodal functionality have allowed lesions in GA to be observed in detail (Figure 5) [163]. GA can be readily visualized in fundus photography as a sharply demarcated area of absent RPE [164]. Without the RPE, the underlying choroidal vessels can be seen [165]. While fundus photography has been the standard method of assessment of GA in the past, newer imaging modalities have replaced fundus photography due to the difficulty in measuring atrophic patches from intersubject variability of fundus pigmentation and the presence of small satellites of atrophy [166,167]. Fundus autofluorescence (FAF) is one of the current standard imaging technologies used for visualizing the RPE in GA [166,168]. Intracellular lipofuscin is found within the RPE, and when atrophy of the RPE occurs, FAF can be utilized to visualize lesions, which appear as distinct dark areas [169,170]. 

The Consensus Definition for Atrophy Associated with Age-Related Macular Degeneration supported the use of OCT imaging as the gold standard for atrophy diagnosis [171]. OCT has been instrumental for diagnosis and treatment, monitoring other retinal diseases such as central serous chorioretinopathy, diabetic macular edema, and macular holes [172,173,174]. Atrophy of different retinal layers and development of choroidal neovascularization can be seen, with OCT providing great prognostic benefit and early identification of potential neovascular AMD [166,175]. Simultaneous recording of cSLO and OCT allows for improved correlation of pathological findings in different imaging modalities [176,177]. The Classification of Atrophy Meeting (CAM) introduced the distinction between complete retinal pigment epithelial and outer retinal atrophy (cRORA) and incomplete RORA (iRORA) based on OCT [178]. cRORA is the endpoint for AMD-related atrophy, while iRORA is an intermediate stage between AMD and GA. OCT-A has also proven useful in the assessment of GA through analysis of the vascular network. GA visualized through OCT-A shows loss of the choriocapillary flow [179,180]. 

Infrared (IR) imaging and multicolor infrared (MC) imaging has also been used frequently in the evaluation of GA. MC is generated by the superimposition of three images acquired simultaneously through blue reflectance (486 nm), green reflectance (518 nm), and infrared reflectance (815 nm). These images are then combined with confocal scanning laser ophthalmoscopy (cSLO) [168,181]. MC has been shown to perform well at evaluating foveal GA, particularly when foveal iRORA is present [168].

### 6.2. Prior Treatments

Prior to complement inhibition therapy, no treatment options were available that halted or reversed the progression of GA. However, physicians generally recommend maintaining a healthy lifestyle and diet to reduce the risk of developing AMD [182]. Studies have shown a link between dietary patterns and the risk of developing AMD [183]. Smoking cessation is also generally advised for all patients, given the association between AMD progression and smoking [182]. While AREDS2 vitamin supplementation decreased the odds of progression to neovascular AMD, the study did not show beneficial effects of slowing down the progression of GA [184,185].

In an attempt to find a therapeutic agent for GA, other studies have looked at the role of neuroprotective agents, anti-inflammatories, and vasodilators in GA [179]. In a phase 2b clinical trial evaluating the use of brimonidine in GA, there was a numeric trend for reduction in GA progression in the intervention cohort compared to the control group [186]. However, the primary efficacy endpoint was not met at 24 months [186]. Ciliary neurotrophic factor (CNTF) is a member of the IL-6 family of cytokines and helps prevent photoreceptor damage. A phase 2 clinical study of CNTF showed a slowdown of visual loss progression at 1-year follow-ups with patients with GA [187,188]. Corticosteroids such as fluocinolone acetonide have also been investigated in clinical trials, but results have not yet been made available [189]. Lampalizumab, a monoclonal antibody, underwent evaluation in two identically designed phase 3 clinical trials, Chroma and Spectri, but did not reduce GA enlargement compared to control during 48 weeks of treatment [150]. Vasodilators such as alprostadil and sildenafil have also been investigated in the treatment of GA. Alprostadil was shown to improve visual acuity in a phase 3 clinical trial and was shown to likely improve BCVA in a meta-analysis of patients with GA [190,191]. 

Another class of drugs that are undergoing extensive research are complement inhibitors. Of note are pegcetacoplan and avacincaptad pegol, two complement inhibitors that became FDA approved for GA in 2023, marking a milestone in GA management [37,192]. The following section will focus on the development, mechanism of action, and clinical effectiveness of these FDA-approved medications. The other complement inhibitors that are undergoing pre-clinical evaluation and clinical trials will be discussed in a later section.

## 7. Pegcetacoplan (SYFOVRE) for Geographic Atrophy

Pegcetacoplan (SYFOVRE) is a complement inhibitor developed by Appelis Pharmaceuticals and is the first FDA approved medication for the treatment of GA in AMD [193,194]. It targets C3 and C3b in the complement system and may also regulate complement-mediated functions [195,196]. For its anti-complement effects, pegcetacoplan has been previously approved by the FDA in 2021 for systemic use in treating paroxysmal nocturnal hemoglobinuria [197]. Due to the role of the complement system in GA, pegcetacoplan has been hypothesized to limit the progression of GA. In the phase 2 FILLY trial, pegcetacoplan was shown to reduce GA lesion growth when compared to control [192]. Subsequently, in the phase 3 OAKS and DERBY trials, the efficacy and safety of pegcetacoplan was evaluated and shown to have an acceptable safety profile [195]. Pegcetacoplan was approved by the FDA in February 2023. Currently, a phase 4 clinical trial is underway to evaluate the real-world safety, tolerability, and treatment patterns of pegcetacoplan. 

### 7.1. Mechanism of Action

As discussed in a prior section, the overactivation of the complement system is strongly linked with the pathogenesis of GA [36]. Dysregulation of the system leads to inflammation and amplification of the alternative complement pathway, which leads to the atrophy seen in GA [198]. Pegcetacoplan binds to complement protein C3 and C3b with high affinity. This regulates the cleavage of C3, affecting the downstream complement pathway. As a C3 inhibitor peptide, pegcetacoplan inhibits C3 convertase, which prevents cleavage of C3 into C3a and C3b [37]. In addition, by binding to C3b, pegcetacoplan prevents C3b’s function in cleaving C5 into C5a and C5b [63]. This prevents inflammatory reactions and activation of the membrane attack complex, leading to cell death. Since C3 is at the convergence of the three complement activation pathways, it is hypothesized to be an ideal target for inhibiting the downstream effects of the complement system [63]. 

### 7.2. Description, Dosing, and Administration

Pegcetacoplan is a pegylated pentadecapeptide with a molecular weight of approximately 43.5 kDa [199]. The solution is a clear, colorless-to-light yellow, aqueous liquid that is contained in a single-dose vial. The vial contains 0.1 mL of solution composed of 15 mg of pegcetacoplan, 5.95 mg of trehalose dihydrate, 0.0895 mg of glacial acetic acid, 0.0353 mg of sodium acetate trihydrate, and water. The solution does not contain an anti-microbial preservative [199]. 

The medication should be stored in the refrigerator between 2 °C to 8 °C and away from light [199]. Once ready, the medication should be warmed to room temperature 15 min prior to preparation. The medication may be loaded into a 1 CC syringe and delivered in a single dose via a 29-guage or 27-guage thin-wall injection needle with Luer lock hub. The standard intravitreal injection anesthetization and sterilization techniques should be practiced throughout the procedure. Intraocular pressure should be checked prior to administration and lowered if necessary. The recommended dosage is 15 mg of pegcetacoplan administered via intravitreal injection to the affected eye once every 25 to 60 days [199].

### 7.3. Phase 2 Clinical Trial (FILLY)

In 2021, results from the phase 2 clinical trial, FILLY, were published [192]. In this 18-month, prospective, multicenter, randomized, sham-controlled phase 2 study, 246 patients were randomized in a 2:2:1:1 ratio, receiving 15 mg pegcetacoplan monthly, 15 mg pegcetacoplan every other month (EOM), sham injections monthly, and sham injections EOM, respectively. Of the 246 patients, 218 completed the first 12 months of the study. Treatment was given for 12 months, and patients were asked to return at 15 and 18 months for safety and efficacy follow-ups. The primary efficacy endpoint was the change from baseline to month 12 in the square root of the GA lesion area, which was assessed using FAF. Secondary outcomes included BCVA, foveal encroachment, and low-luminance BCVA [192]. 

The efficacy endpoint was met in both treatment groups with a least square mean change of 0.25 mm in the monthly group, 0.28 mm in the EOM group, and 0.35 mm in the pooled sham group [192]. Compared to the sham group, the monthly group and EOM group showed a 30% smaller increase and a 20% smaller increase in total GA lesion growth over a 12-month period. The majority of this difference occurred between months 6 and 12 of the study. All cohorts experienced a gradual decline in visual acuity and low-luminance BCVA with no significant difference between groups. There was a higher rate of adverse events, such as choroidal neovascularization or neovascular AMD in the monthly pegcetacoplan and EOM pegcetacoplan groups, 20.9% and 8.9%, respectively, compared to the control group (1.2%). Development of these adverse events were not associated with a substantial change in BCVA [192]. 

A post hoc analysis was conducted to investigate this observed dose-dependent increase in new-onset exudative AMD [200]. Results from the study showed an increased rate of baseline DLS and history of exudative AMD in fellow eyes compared to the natural prevalence. It was also suggested that the single masked nature of the FILLY trial may have led to over-reporting in the treatment group. Complement-mediated effects leading to neovascularization were also suggested. In a separate post hoc analysis study, researchers found eyes receiving pegcetacoplan to have lower rates of progression from iRORA to cRORA compared to controls [201]. 

### 7.4. Phase 3 Clinical Trial (OAKS and DERBY)

The OAKS and DERBY trials were 24-month, multicenter, randomized, double-masked, sham-controlled, phase 3 clinical studies that were designed for regulatory purposes [195]. Similarly to the FILLY trial, a 2:2:1:1 ratio of 15 mg pegcetacoplan monthly, 15 mg pegcetacoplan EOM, sham injection monthly, and sham injection EOM was used to assign treatment groups. Groups of 637 and 621 patients were enrolled, of which 471 and 469 of the patients completed all 24 months of the study in the OAKS and DERBY trials, respectively. Patients included were required to have a total GA area of 2.5–17.5 mm2, and, if multifocal, at least one lesion with an area of 1.25 mm2 was identified on FAF. Patients were treated and monitored for 24 months. In addition to standard FAF imaging and BCVA assessment, fluorescein angiography was obtained for all patients at baseline, month 12, and month 24. The primary endpoint was the change from baseline to month 12 in the total area of GA evaluated via FAF. Other secondary outcomes included the monocular maximum reading speed, BCVA, and the change in mean threshold sensitivity evaluated via mesopic microperimetry (OAKS only) [195]. 

In the OAKS trial, the primary endpoint was met for both treatment groups at 12 and 24 months, showing a slowed rate of growth of GA compared to the sham group [195]. The monthly and EOM injection group showed a 21% and 16% slower rate of growth at 12 months, respectively. At 24 months, a 22% and 18% slower rate of growth was observed in the monthly and EOM injection groups. These results were statistically significant. In the DERBY trial, the primary endpoint was not met at 12 months. The GA rate of growth was slower by 12% and 11% compared to the sham group at 12 months for the monthly and EOM injection groups, respectively. This was not statistically significant. At 24 months, the monthly and EOM injection groups showed a significant slowing of GA progression by 19% and 16%, respectively. In non-subfoveal GA lesions, growth was slowed by 26% and 22% for the monthly and EOM groups, respectively. In subfoveal GA lesions, growth was slowed by 19% and 16% for the monthly and EOM groups respectively. There were no significant differences between treatment groups and sham in visual function measurements and mean threshold sensitivity at 24 months [195]. 

In both trials combined, the rate of intraocular inflammation was 0.22% per injection at 12 months [195]. Four cases of intraocular inflammation were observed and linked to drug impurity. No events of permanent, severe vision loss were reported. Four cases of infectious endophthalmitis with return to baseline visual acuity were reported, as were three cases of ischemic optic neuropathy. New onset exudative AMD was reported in 11%, 8%, and 2% of patients in the monthly, EOM, and sham groups, respectively, at 24 months in the OAKS trial. These rates were 13%, 6%, and 4% in the monthly, EOM, and sham groups, respectively, for the DERBY trial. Patients with newly diagnosed exudative AMD continued their treatment regiments and received anti-VEGF therapy in 98%, 96%, and 85% of patients in the monthly, EOM, and sham groups respectively. Unexpected outcomes were not observed in patients who received pegcetacoplan with anti-VEGF treatment [195]. Table 3 summarizes the clinical data in the FILLY, OAKS, and DERBY trials regarding reduction in GA growth in pegcetacoplan therapy. 

### 7.5. Side Effects, Contradictions, and Reception

Pegcetacoplan is contraindicated in patients with active intraocular inflammation, ocular infections, or periocular infections [199]. As with all intravitreal injections, pegcetacoplan may be associated with endophthalmitis and retinal detachments. In both clinical trials, pegcetacoplan was associated with increased rates of neovascular or exudative AMD and appeared to follow a dose-dependent manner [202]. Patients undergoing pegcetacoplan therapy should therefore be monitored for signs of neovascular AMD and receive anti-VEGF therapy if needed [199]. Another feared complication includes ischemic optic neuropathy, although the cause of this complication is unknown [202]. Other common adverse effects include intraocular inflammation, eye discomfort, conjunctival hemorrhage, posterior capsule opacification, punctate keratitis, vitreous floater, increased intraocular pressure, and visual impairment [195,199,202]. Retinal vasculitis with or without retinal vascular occlusion was also reported during postapproval use of pegcetacloplan [199].

As the first FDA approved drug for GA, pegcetacoplan was received with both excitement and a fair amount of caution, leading to varying levels of adoption across providers. Some of the concerns included the lack of improvement in visual outcomes in patients as well as the higher risks of complications, particularly exudative AMD [203]. Both phase 3 clinical trials, as well as the phase 2 clinical trial, showed increased rates of exudative AMD in patients [192,195]. Therefore, while the medication may reduce the rate of growth of GA, some argue that the potential risks of the medication outweigh the benefits [203,204]. Another concern included the drop out rate for patients prior to the 24-month analysis. In the OAKS trial, 31% of patients dropped out of the study before month 24 [195]. Further clinical studies should be conducted to better understand the adverse outcomes of pegcetacoplan as well as further clinical uses in the management of GA. 

## 8. Avacincaptad Pegol (IZERVAY) for Geographic Atrophy

Avacincaptad pegol (IZERVAY, formerly Zimura) is another complement inhibitor developed by Astellas Pharma Inc. that became the second treatment approved by the FDA for the treatment of GA [146,202]. The primary target of the drug is factor C5 in the complement system. Similar to pegcetacoplan, avacincaptad pegol has been hypothesized to limit the progression of GA by inhibiting the complement system [64]. Two clinical studies, GATHER1 and GATHER2, evaluated the efficacy and safety of avacincaptad pegol and found a decrease in the rate of progression in GA compared to sham at 12 months [204]. Neither study saw increased rates of serious adverse ocular events. Following completion of the clinical trials, the drug was approved by the FDA in August of 2023, making it the second treatment approved for use on GA [202].

### 8.1. Mechanism of Action

The dysfunction of the complement system is linked to the pathogenesis of GA. As a potent and specific inhibitor of complement C5, avacincaptad pegol prevents cleavage of C5 and formation of terminal fragments, C5a and C5b [64]. C5a serves an important role in inflammasome priming and activation, leading to cell death [205]. C5b helps with the formation of the membrane attack complex, which also leads to cell death [206]. By targeting the downstream factors in the complement cascade, avacincaptad pegol limits irregular apoptosis while preserving the earlier steps of the complement system. Unlike with pegcetacoplan, this would theoretically preserve the neuroprotective and anti-inflammatory effects of C3 and C3a [204,207]. The goal of complement inhibition in GA is to slow down the progression of atrophy and achieve therapeutic benefits [64]. 

### 8.2. Description, Dosing, and Administration

Avacincaptad pegol is a ribonucleic acid aptamer that is bound to a branched polyethylene glycol molecule [208]. It weighs approximately 56 kDa. It is a clear to slightly opalescent, colorless to slightly yellowish solution that is contained in a single-dose vial. The vial contains 0.1 mL of solution, comprising 2 mg avacincaptad pegol, 0.198 mg of dibasic sodium phosphate heptahydrate, 0.0256 mg monobasic sodium phosphate monohydrate, and 0.83 mg sodium chloride and water. The solution does not contain an anti-microbial preservative [208]. 

The medication should be stored in the refrigerator between 2 °C to 8 °C and away from light [208]. Once ready, the medication should be warmed to room temperature and may be kept at room temperature for up to 24 h. The medication may be loaded into a 1 CC syringe and delivered in a single dose via a 30-guage × ½ inch injection needle. The standard intravitreal injection anesthetization and sterilization technique should be practiced throughout the procedure. Intraocular pressure should be monitored following intravitreal injection. The recommended dosage is 2 mg of avacincaptad pegol, administered via intravitreal injection to the affected eye once every 28 ± 7 days for up to 12 months [208].

### 8.3. Phase 2/3 Clinical Trial (GATHER1)

In 2021, results from the phase 2/3 clinical trial, GATHER1, were published [64]. This was a 12-month, prospective, multicenter, randomized, double-masked, sham-controlled phase 2/3 study with two parts. Groups of 77 and 209 patients were enrolled in part one and part two, respectively. In part one, patients were randomized in a 1:1:1 ratio to form three cohorts: 1 mg of avacincaptad pegol, 2 mg of avacincaptad pegol, and sham. In part two, patients were randomized in a 1:2:2 ratio to form three cohorts: 2 mg of avacincaptad pegol, 4 mg of avacincaptad pegol, and sham. In part two, all patients received two injections due to drug viscosity. Patients received monthly injections for 12 months in both parts. As opposed to the FILLY, OAKS, and DERBY trials, patients with evidence of CNV in the fellow eye were excluded in this study [192,195]. The primary efficacy endpoint was the mean rate of change in the square root of the total GA area over 12 months. Secondary outcomes included BCVA and low luminance BCVA [64]. 

Efficacy endpoints were met in both the avacincaptad pegol 2 mg and avacincaptad pegol 4 mg groups, reducing the mean GA progression over 12 months [64]. The mean growth rate of total GA size was reduced by 30.5% and 25.6% compared to sham treatment in the 2 mg and 4 mg treatment groups, respectively. Results from the 1 mg avacincaptad pegol cohort were not part of the prespecified statistical analysis but also showed reduction in GA progression compared to sham. All cohorts experienced a decrease in visual acuity, but there was no difference in mean change in BCVA or low luminance BCVA from baseline to month 12. Choroidal neovascular membrane formation was reported in the fellow eyes of 10 patients. In the study eyes, investigators observed choroidal neovascular membrane formation in 2.7% of the sham cohort, 4.0% of the 1 mg cohort, 9.0% of the 2 mg cohort, and 9.6% of the 4 mg cohort. Unlike the pegcetacoplan trials, patients who developed CNV were discontinued from the study [64]. 

In a continuation of the GATHER1 study, results for 18 months of treatment were recently reported [209]. Patients continued to receive their respective avacincaptad pegol doses, as previously described, for an additional 6 months. During this 6-month period, further separation between the treatment cohorts and sham cohort was observed. GA progression was reduced by 32.2% and 29.4% in the 2 mg and 4 mg cohort respectively. Visual acuity continued to decline throughout the observed period. However, patients receiving avacincaptad pegol experienced better outcomes in BCVA and low luminance BCVA compared to sham groups. Macular neovascular conversion rates were 11.9%, 15.7% and 2.4% in the 2 mg, 4 mg, and sham groups, respectively. Again, these eyes were discontinued from the study upon observation of macular neovascular conversion [209]. 

### 8.4. Phase 3 Clinical Trial (GATHER2)

Results from the first 12 months of the GATHER2 trial were recently published following the FDA approval of avacincaptad pegol [203]. The study was a prospective, randomized, double-masked, sham-controlled phase 3 clinical trial. An amount of 448 patients were assigned in a 1:1 ratio of receiving monthly avacincaptad pegol 2 mg or sham. Avacincaptad pegol 2 mg was used as the treatment arm as opposed to 4 mg due to the favorable benefit-risk profile observed in the GATHER1 study. The primary efficacy endpoint was the square-root of the total GA area growth from baseline to month 12. The secondary endpoint included BCVA and low luminance BCVA. Unlike in the GATHER1 study, patients who developed macular neovascularization remained in the trial and received appropriate anti-VEGF treatment [204]. 

The primary endpoint was met, and the data showed a reduction in the mean rate of GA growth at 12 months compared to sham [204]. The rate of growth of the total GA lesion area without square root transformation was reduced by 18% in the 2 mg treatment arm compared to sham. There were no statistically significant differences in BCVA and low-luminance BCVA change between the treatment arm and sham arm. Notably, 7% of patients in the avacincaptad pegol 2 mg group and 4% of patients in the sham group developed macular neovascularization in the study eye. There were no events of endophthalmitis or ischemic optic neuropathy in either group over the 12 months [204]. Table 4 summarizes the reduction in GA growth in the GATHER1 and GATHER2 trials.

### 8.5. Side Effects, Contradictions, and Reception

Avacincaptad pegol is contraindicated in patients with active intraocular inflammation, ocular infections, or periocular infections [208]. Similar to pegcetacoplan and intravitreal injections, avacincaptad pegol may be associated with endophthalmitis and retinal detachments [208]. Patients should also be monitored for neovascular AMD and CNV, as both clinical trials showed increased rates of macular neovascularization conversion, primarily of the type 2 subtype [64]. The percentage of exudative macular neovascularization was 5% in patients receiving avacincaptad pegol 2 mg and 3% in the sham group in the GATHER2 trial [204]. Patients who develop this complication should be further evaluated to receive appropriate anti-VEGF therapy if there are no contraindications. Other common adverse effects include conjunctival hemorrhaging, increased IOP, vision impairment, eye discomfort, vitreous floaters, and blepharitis [204,208]. 

The approval of avacincaptad pegol by the FDA furthered an already exciting year for GA treatment. However, similar to the reception of pegcetacoplan, avacincaptad pegol was received with caution amongst providers. While rates of exudative transformation were lower in these clinical trials, they still posed a potential downside when considering new therapeutic agents for patients [202,204]. Visual outcomes were not improved in patients either, which again calls into question the practicality of these medications [202]. As such, future research and understanding in the field of complement inhibition can help stimulate development of new therapeutics in GA and methods for countering adverse effects. 

## 9. Conclusions

Inhibition of the complement system as a therapeutic target is an area of high interest in the field of retinal treatment. From in vitro models to clinical trials, there have been ongoing developments regarding insights into mechanisms, benefits, contraindications, and side effects. Further research is required to gain additional insights into the clinically available medications for GA, including longitudinal phase 4 trials. Additional research regarding complement inhibition in other retinal diseases also serves as an area of critical research. Ultimately, the complement pathway is a complex, unique system that plays a critical role in retinal health and disease, thus serving as an important topic for research.

## Figures and Tables

**Table 1:** Pre-clinical studies, including genetic, in vitro, and animal models, investigating the complement system in retinal disease.

Investigation	Experimental Model	Main Findings	Representative Reference
C1-esterase inhibition	Chinese hamster ovary in vitro model; rat model	Modest complement inhibition with concentration-dependent cytotoxicity; decreased retinal vascular permeability	[106,140]
Anti-C1s antibody (TNT009)	In vitro alloserum from dialysis patient; in vitro RBCs	Decreased activation of complement pathway	[87,141]
Kallikrein inhibition	Rat model	Decreased retinal vascular permeability	[106]
C3 inhibition/absence	In vitro RPE cells; Primate/murine model	Restored lysosomal function and reversed disease phenotype; suppressed drusen formation; decreased retinal degeneration	[90,110,112]
C3a/C3a receptor inhibition	In vitro embryonal chick cells; in vitro human fetal RPE cells; murine model	Retinal regeneration; C3ar blockage prevents C3a-induced sub-RPE deposit formation; supplementation associated with increased proteasome activity; alleviated neuroinflammation and restored visual function with receptor blockade	[91,117]
C5 inhibition	In vitro RPE cells; murine model	Suppressed priming for inflammasome activation; inhibited choroidal neovascularization after laser; improved retinal function, decreased loss of retinal ganglion cells, cones, and photoreceptors; preserved optic nerve function	[95,118,121,122,123]
C5a/C5a receptor inhibition	Murine model	C5a inhibition insufficient to block development of AMD-like pathology; C5ar-mediated signaling key to normal retinal function and structure; C5ar-deficient mice have increased neovascularization	[120,125,127]
CD59	Murine model	Inhibited growth and reduced size of choroidal neovascularization	[60]
Factor B inhibition/absence	In vitro RPE cells; In vitro erythrocytes; Murine model	Blocking TNF-α can block factor B production; factor B inhibition inhibits alternative pathway; normalized dysregulated complement pathway, reduced sub-RPE deposits with inhibition	[97,99,130]
Factor D inhibition/absence	In vitro erythrocytes; Murine model	Alternative pathway blockade preserves bacterial clearance; photoreceptor protection	[133]
Factor H	In vitro purified C3b; Rat/murine models	Factor H competition results in complement dysregulation; complete knockout associated with increased retinal inflammation and amyloid β deposition	[25,105,128]
Factor I	Murine model	Reduction in choroidal neovascularization	[138,139]

RPE, retinal pigment epithelium; RBC, red blood cells.

**Table 2:** Clinical trials targeting the complement system in retinal disease, including the different phases and status.

Pharmaceutical	Target	Status	Reference/Trial Identifier
Cinryze	C1 esterase inhibitor	Phase 1b completed (2014), current status unknown	144; NCT01759602
NGM621	C3 inhibitor	Phase 2 completed (2023)	NCT04465955
POT-4	C3 inhibitor	Phase 2 terminated	NCT01603043
Avacincaptad Pegol/ARC1905/Zimura	C5 inhibitor	Various stages	NCT03374670; NCT03364153; NCT03362190; NCT05536297
Eculizumab	C5 inhibitor	Phase 2 completed	NCT00935883
LFG316/Tesidolumab	C5 inhibitor	Phase 2 completed	NCT01255462; NCT01526889
IONIS-FB-LRx	Factor B inhibitor	Phase 2 ongoing	NCT03815825
FCFD4514S/Lampalizumab	Factor D inhibitor	Phase 2 completed, terminated	NCT02288559
ALXN2040/Danicopan	Factor D inhibitor	Phase 2 ongoing	NCT05019521
CLG516	Properidin inhibitor	Phase 2 completed (2019)	NCT02515942
GT005	AAV-based Factor I gene therapy	Phase 2 terminated	NCT04566445

AAV, adeno-associated virus.

**Table 3:** Clinical trial data on reduction in geographic atrophy growth compared to the combined sham group for pegcetacoplan therapy from the FILLY, OAKS, and DERBY trials. Percentages reported as differences in the total GA area.

	Reduction in GA Growth Compared to Combined Sham Group		
Treatment	FILLY (Phase 2)	OAKS (Phase 3)	DERBY (Phase 3)
12 Months			
Monthly Injection	30%	21%	12%
EOM Injection	20%	16%	11%
24 Months			
Monthly Injections	-	22%	19%
EOM Injections	-	18%	16%

Every other month, EOM; Geographic atrophy, GA.

**Table 4:** Clinical trial data for pegcetacoplan therapy in reducing geographic atrophy growth compared to the sham group in the GATHER1/2 trials. Percentages reported as differences in total GA area.

	Reduction in GA Growth Compared to Sham Group	
Treatment	GATHER1 (Phase 2/3)	GATHER2 (Phase 3)
12 Months		
2 mg	30.5%	18%
4 mg	25.6%	-
18 Months		
2 mg	32.2%	-
4 mg	29.4%	-
